# Efficacy of sealants and bonding materials during fixed orthodontic treatment to prevent enamel demineralization: a systematic review and meta-analysis

**DOI:** 10.1038/s41598-021-95888-6

**Published:** 2021-08-16

**Authors:** R. Kamber, H. Meyer-Lueckel, D. Kloukos, C. Tennert, R. J. Wierichs

**Affiliations:** 1 Department of Restorative, Preventive and Pediatric Dentistry, zmk Bern, University of Bern, Freiburgstrasse 7, 3010 Bern, Switzerland; 2 Department of Orthodontics and Dentofacial Orthopedics, zmk Bern, University of Bern, Bern, Switzerland

**Keywords:** Caries sealants, Fixed appliances

## Abstract

To analyse clinical studies investigating coating agents such as sealants and other bonding materials to prevent the initiation or inhibit the progress of white spot lesions (WSL) during orthodontic treatment with fixed appliances. Electronic databases (Pubmed, CENTRAL, EMBASE) were screened for studies. No language restrictions were applied. Study selection, data extraction and quality assessment were done in duplicate. Primary outcome included assessment of WSL with visual-tactile assessment and/or laser fluorescence measurements. Twenty-four studies with 1117 patients (age: 11–40 years) and 12,809 teeth were included. Overall, 34 different sealants or bonding materials were analysed. Fourteen studies analysed fluoride and 14 studies non-fluoride releasing materials. Meta-analysis for visual tactile assessment revealed that sealants significantly decreased the initiation of WSL compared to untreated control (RR [95%CI] = 0.70 [0.53; 0.93]; very low level of evidence). Materials releasing fluoride did not decrease initiation of WSL compared to those with no fluoride release (RR [95%CI] = 0.84 [0.70; 1.01]; very low level of evidence). For laser fluorescence measurements no meta-analysis could be performed. The use of sealants seems to be effective in preventing the initiation of post-orthodontic WSL. Furthermore, there is no evidence supporting that fluoride-releasing sealants or bonding materials are more effective than those without fluoride release. No gold standard prevention strategy to prevent WSL during treatment with fixed orthodontic appliances has been established yet. However, based on only a limited number of studies the use of sealants seems to be effective in preventing the initiation of post-orthodontic WSL.

## Introduction

Contemporary orthodontic treatment with fixed appliances is known to be effective in resolving teeth crowding and levelling the dental arches. However, fixed orthodontic appliances might be associated with adverse effects on enamel, due to plaque accumulation and their colonisation by oral microbes. Moreover, due to impaired oral hygiene, the risk of carious lesions development is increased^1–3^. Due to their whitish, opaque or chalky appearance, caused by mineral loss in enamel^4^, these lesions are often termed as white spot lesions (WSL).

WSL develop quickly^5^ and are often an aesthetic burden to the patients even years after removal of orthodontic appliances^6,7^. The prevalence after treatment with fixed orthodontic appliances has been reported to vary between 11%^8^ and even 97%^9^. Furthermore, incidence of WSL among patients treated with fixed appliances has been reported between 7%^10^ and 73%^11^. Although, this wide range might be explained by the inconsistent definitions and settings in the studies and the different WSL scores^4^, the incidence of WSL among patients treated with fixed appliances is consistently and significantly higher than among patients without any orthodontic treatment^3^.

Once the brackets have been removed, WSL may remineralize in case of improved oral hygiene. Application of fluoride containing agents^12,13^, acidulated phosphate fluoride (APF)^14^ or casein phosphopeptides-amorphous calcium phosphate (CPP-APP) containing pastes can be used to enhance remineralization. However, the aesthetic appearance in most cases may remain impaired^15,16^. Although micro-invasive treatment options can successfully recover the aesthetic appearance by masking these lesions^17,18^, various preventive strategies during treatment with fixed appliances have been employed to prevent the initiation of WSL. Patients were instructed to use fluoride-, chlorhexidine- or CPP-ACP-containing products^19,20^. However, all of these preventive strategies mostly depend on the patients’ compliance that may not always be adequate^21^. Thus, it is most likely that these strategies fail to completely prevent the development of WSL^22^.

Another preventive strategy, which does not depend on the patients’ compliance, is the use of coating agents, namely sealants or other bonding materials. These are applied by dentists directly before^23^, during^24^ or after^25^ the bonding of fixed appliances. They are supposed to build a diffusion barrier or decrease enamel demineralization around orthodontic brackets by releasing fluorides. Some of these interventions have been shown to reduce the incidence of WSL^23,26,27^. Indeed, two systematic reviews revealed that sealants were associated with reduced WSL incidence than no sealant^28,29^. However, only up to eight studies were included in each systematic review although there are more clinical studies that analyse coating agents.

Thus, the aim of this systematic review was to evaluate the efficacy of various (resin-based) coating materials to prevent initiation and reduce the progression of WSL during orthodontic treatment with fixed appliances. Null hypothesis was that no difference between coating versus no coating, or between coating with versus without fluoride release can be observed.

## Material and methods

### Review design

No study registration is necessary for this review. The Preferred Reporting Items for Systematic Reviews and Meta-Analyses (PRISMA) were adopted throughout the process of the present systematic review^30^. The present review aimed at systematically retrieving and analysing clinical studies investigating sealants and bonding materials used in orthodontic therapy with fixed appliances to prevent initiation or decelerate the progression of WSL.

A literature search was performed, and study inclusion followed predefined criteria. The following data were extracted: Initiation as well as progression/regression of WSL after the use of.

- sealants (being applied before or after the brackets are inserted) as well as
- bonding materials (being applied while the brackets are inserted)

being assessed by different diagnostic methods (e.g. visual-tactile methods, laser fluorescence measurements, etc.). Although analyses indicated that sealants or bonding materials (with/without fluoride release) may be capable to reduce the progression of WSL, meta-analyses were planned for agents with similar intervention and outcome measures investigated in more than one study.

According to the PICOS (Population, Intervention, Outcome, Study design) scheme, prospective controlled randomized clinical trials and non-randomized controlled or studies on human patients assessing the effect of any kind of fixed orthodontic appliances on WSL were included^31^ (Table 1).

**Table 1:** PICOS schema: Population (P), Intervention (I), Comparison (C), Outcomes (O) and Study Design (S).

P—Participants: patients of any age receiving comprehensive orthodontic treatment
I—Intervention: treatment with fixed appliances using a coating material (sealant or bonding) with or without fluoride release
C—Control: teeth with brackets without additional sealing or such treated with a non-fluoride containing material
O—Outcome: development (initiation and progression/regression) of demineralization (WSL), visual-tactile assessment and DIAGNOdent assessment
S—Studies: RCTs and non-randomized controlled clinical studies

### Inclusion and exclusion criteria

The following inclusion criteria were adopted:

- Clinical study on orthodontic treatment with multi-bracketed fixed appliances (no further specification regarding e.g. minimum follow-up period, minimum number of participants, etc. were made)
- assessment of different coating materials (sealants and bonding materials)
- assessment of WSL (e.g. visual tactile assessment, laser fluorescence measurement, etc.)

The following exclusion criteria were adopted:

- in vitro studies, animal studies, editorials, reviews
- outcomes not assessing WSL
- ‘single group studies’/clinical studies without any control group

### Search strategy

Detailed search strategies were developed and appropriately revised for each database, considering the differences in controlled vocabulary and syntax rules by the first author (R.K.). Database searches were performed in Pubmed, CENTRAL and Embase without limitations in language or year of publication. The search strategy for the 3 major databases is shown in Supplementary Table S1. The reference lists of all identified eligible studies and other published systematic reviews were hand-searched in order to identify further eligible studies.

Two authors (R.K. and R.J.W.) independently reviewed the titles and abstracts of articles retrieved by the use of a defined search strategy (Supplementary Table S1). The reviewers were not blinded to journal names or article authors. A detailed sequence of filtering search results to include relevant articles can be found in the Supplementary document.

### Study selection

Study selection was performed independently and in duplicate by two authors of the review (R.K. and R.J.W.), who were not blinded to the identity of the authors of the studies, their institutions, or the results of their research. Study selection procedure comprised of title-reading, abstract-reading and full-text-reading stages. After exclusion of non-eligible studies, the full report of publications considered by either author as eligible for inclusion was obtained and assessed independently. Disagreements were resolved by discussion and consultation with the third author of the review (C.T.). A record of all decisions on study identification was kept.

### Data extraction

Two authors (R.K. and R.J.W.) extracted the data by means of predefined structured tables. Data extraction was performed independently and in duplicate. For longitudinal studies or clinical trials published sequentially in different journals only the most recent report was deemed as eligible for inclusion. Unpublished data were not sought from authors or obtained from other sources. For each study, the following data were extracted:

- Study type and setting
- Test and control group
- Design of control
- Type of intervention: coating material (i.e. sealants, bonding materials)
- Product name
- Follow-up
- Primary and secondary outcomes
  - WSL assessment (visual-tactile and laser fluorescence)
  - Oral health indices (e.g. plaque index or gingival bleeding index)

- Number of patients and teeth
  - At the beginning
  - At the end

For missing information, the corresponding author was contacted by e-mail.

### Risk of bias assessment

Two authors (R.K. and R.J.W.) independently evaluated the risk of bias of the included studies. Any disagreement between the reviewers was discussed until an agreement was reached and if needed, by consulting a third author (C.T.). To assess risk of bias the guidelines by the Cochrane Collaboration were used, for non-randomised controlled trials (non-RCT) the ROBINS-I-tool^32^ and for randomised controlled trials (RCT) the Risk of Bias 2.0. tool^33^.

### Data analysis and grading

The statistical analyses were conducted in Review Manager (RevMan version 5.4 software, Cochrane Collaboration, Copenhagen, Denmark, 2014). Statistical significance was defined as a *p*-value ≤ 0.05 (Z test) and heterogeneity was assessed with I^2^^34^. Fixed or random-effects meta-analysis was performed depending on heterogeneity (I^2^ < 35%: fixed-effects; I^2^ > 35%: random-effect)^35^. The number of events was considered as the number new diagnosed WSL. To avoid unit-of-analysis errors the guidelines outlined by the Cochrane collaboration (chapter 9.3.4.) were followed^36^. Therefore, baseline data were compared with data of a single time point (mostly longest follow-up period). Forest plots were created to illustrate the meta-analysis. Grading of evidence was performed according to the GRADE network levels using Grade Profiler 3.6^37^.

### Heterogeneity

Clinical and methodological heterogeneity were assessed by examining the characteristics of the studies, the similarity between the types of participants, the interventions, and the outcomes as specified in the inclusion criteria for considering studies for this review.

Statistical heterogeneity would have been assessed using a Chi^2^ test and the I^2^ statistic, where I^2^ values over 50% indicated substantial heterogeneity.

### Assessment of reporting bias

In the presence of more than 10 studies in a meta-analysis, the possible presence of publication bias was investigated for the primary outcome. Publication bias was assessed by Funnel plots^38^.

### Sensitivity analysis

We explored whether or not the analysis of studies stratified by (1) risk of bias or (2) study design yielded similar or different results. For this (1) studies at high risk of bias or (2) studies using a parallel-arm design were eliminated in a second/third analysis.

## Results

**Figure 1:**
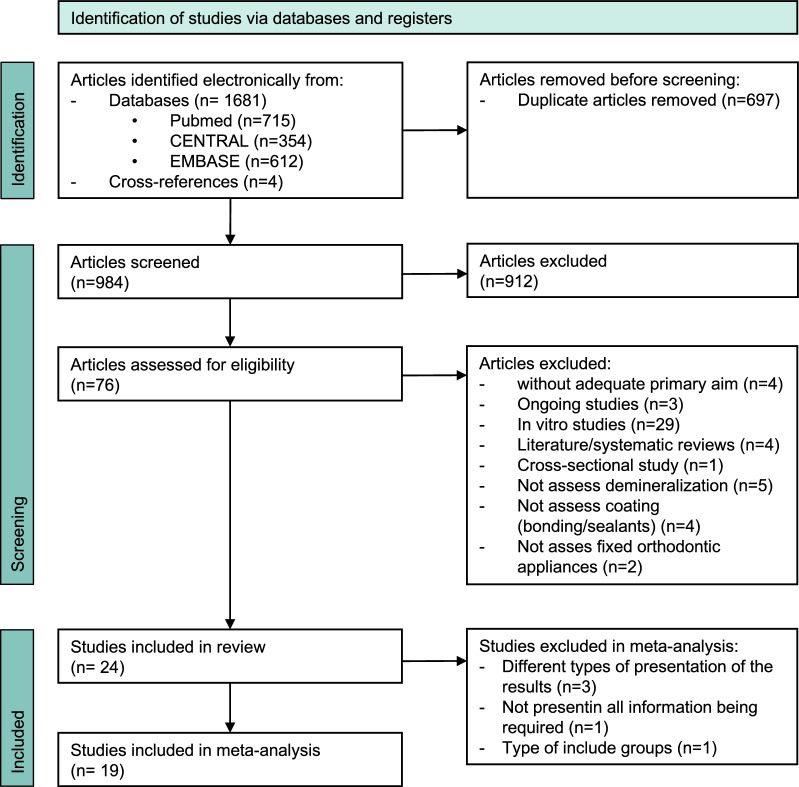
Prisma flow diagram.

After removing 697 duplicates, 984 articles were identified by screening the electronic databases. Further four studies were identified by other sources (e.g. cross references). Seventy-six articles were assessed for eligibility and screened full-text. Overall 52 had to be excluded (Supplementary Table S2) and a total of 24 papers reporting 24 trials were included (Supplementary Table S3, Fig. 1). Thirteen were RCTs, out of which, where 11 studies used the split-mouth design and the other two a parallel-arm design^23,39^. Eleven of the included studies were non-RCTs, out of which 9 studies used the split-mouth design and the other 2 a parallel-arm design^27,40^. Eventually, 1117 patients (age range: 11–40 years) with at least 12,809 teeth being treated were included. Unfortunately, the exact number of treated teeth were not reported in 4 studies^23,27,41,42^. Overall, 34 different coating materials have been analysed (Supplementary Table S4). Median (25th/75th percentiles) follow-up time was 12.65 (6/16.9) months.

**Figure 2:**
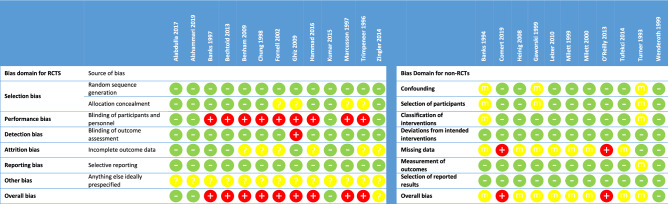
Risk of bias of included studies. RCTs: − , low; + , high; ?, unclear. Non-RCTs: − , low; + , high; m, moderate.

Risk of bias was assessed for all 24 studies; overall risk of bias was low for 4 studies^1,21,43,44^, moderate for 9 studies^27,45–51^, high for 11 studies^23–26,40–42,52–55^ and unclear for 1 study^39^ (Fig. 2).

Initiation and inactivation of WSL were described using visual-tactile assessments (all studies) and additionally by using laser fluorescence (DIAGNOdent) in six studies^1,21,26,40,42,43^. Thirteen studies were randomized controlled trials (RCT)^1,21,23–26,39,42,43,52–55^, and the other 11 were non-randomized controlled studies^27,40,41,44–51^. Thirteen studies tested bonding materials and 11 studies sealants. Most of the studies (n = 12) investigated initiation of new WSL, one study analysed the progression/regression of existing WSL^46^, and 11 studies analysed both^1,21,25,42,43,47,48,50,51,53,55^. A detailed summary of included studies can be found in the Supplementary Table S3.

Although WSL were described by using visual-tactile assessment and laser fluorescence (DIAGNOdent), meta-analyses were only performed for visual-tactile assessments. Analyses could not be performed for laser fluorescence, since 3 of the 6 studies compared sealant vs. no sealant^21,26,42^ and 3 studies compared coating with and without fluoride release^1,40,43^. Furthermore, for the first comparison (coating vs. no coating) results were presented parametrically or non-parametrically not reporting all information required for recalculation. For the second comparison (coating with or without fluoride release) results were either presented using continuous^1,43^ or categorial^40,43^ results. Continuous results were presented with odds ratios^43^ or with means and standard deviations^42^. Categorial results were presented using different (sub)classifications and in one study values of DIAGNOdent and of visual-tactile assessment did not match^43^. Using visual-tactile assessment no WSL were diagnosed in 506 teeth, but all teeth presented a WSL using laser fluorescence assessment^43^. Since not all information—being necessary for recalculation—were available in the mentioned studies, a meta-analysis was not performed. Furthermore, analysis on oral health indices were not possible, since oral health were described by using too many different indices and since in some factors results of oral health indices were only be present for baseline evaluation but not for any follow-ups.

Thus, meta-analysis was only performed for visual-tactile assessments. Although WSL were classified in accordance to different scores (e.g. Gorelick score, modified Gorelick score, DMFT/DMFS) 19 studies reported the presence or absence of WSL. Six of these studies compared the efficacy of coating (with or without fluoride release) compared to no coating^23,25,26,41,44,45^, 10 studies compared coating with and without fluoride release^24,40,43,46,47,49,50,52–54^ and 3 studies compared glass ionomer cements with resin adhesives^51,55^.

**Figure 3:**
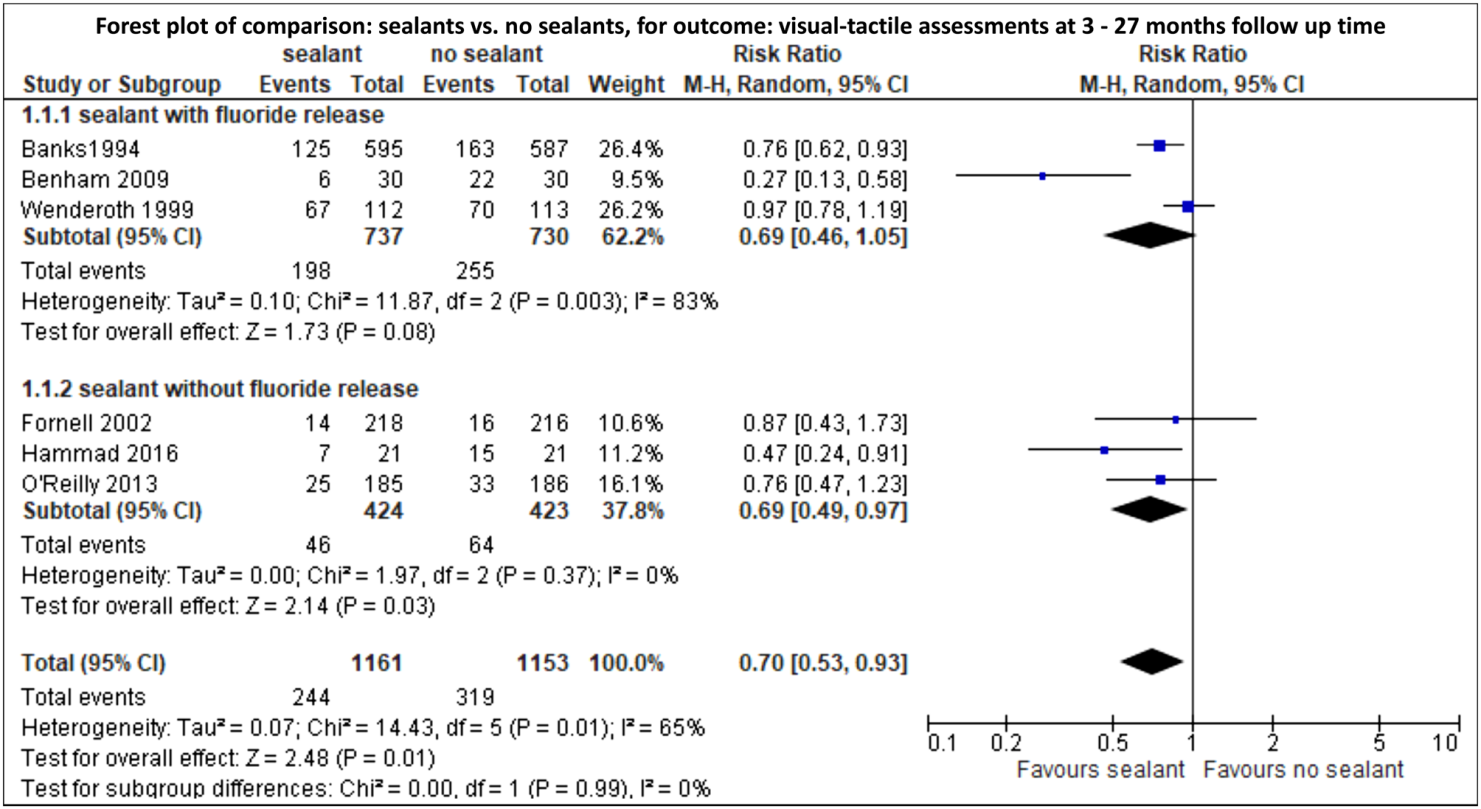
Meta-analysis for the comparison: sealant vs. no sealant. Visual-tactile assessments were used to calculate RR and 95%CI.

**Figure 4:**
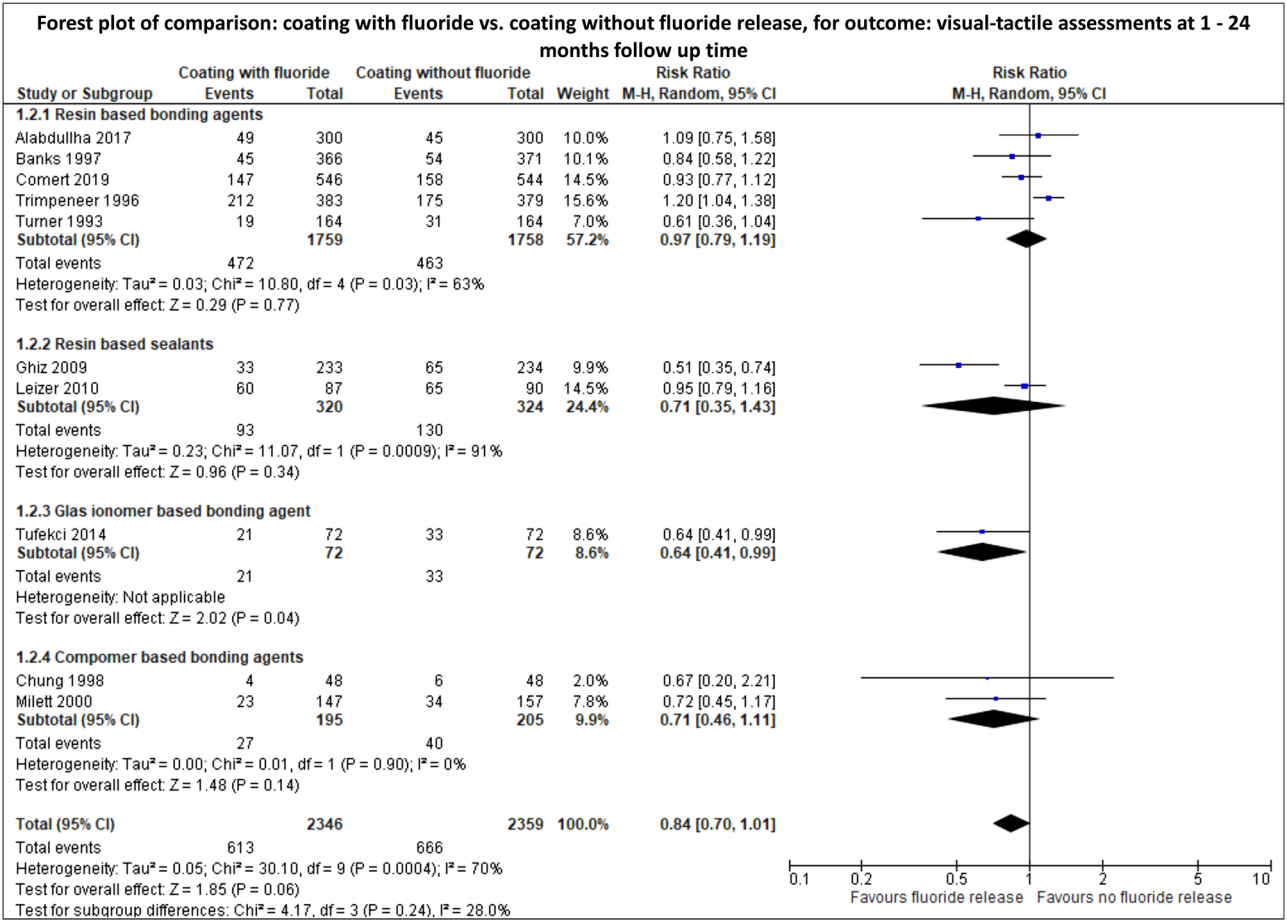
Meta-analysis for the comparison: coating with and without fluoride release. Visual-tactile assessments were used to calculate RR and 95%CI.

**Figure 5:**
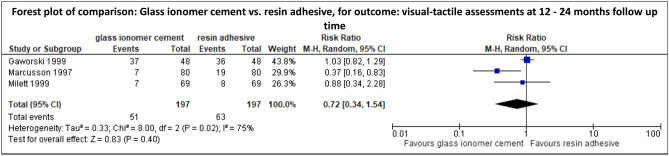
Meta-analysis for the comparison: glass ionomer cement vs. resin adhesive. Visual-tactile assessments were used to calculate RR und 95% Cl.

Meta-analysis revealed that the incidence of WSL was significantly lower after the use of a sealant compared with no additional use of a sealant (RR [95%CI] = 0.70 [0.53; 0.93]; very low level of evidence) (Fig. 3, Supplementary Table S5) and that coating with fluoride release non-significantly decreased the initiation of WSL compared with coating without fluoride release (RR [95%CI] = 0.84 [0.70; 1.01]; very low level of evidence) (Fig. 4, Supplementary Table S5). Furthermore, no significant difference in the incidence of WSL could be observed between the usage of a glass ionomer cement and resin adhesives (RR [95%CI] = 0.72 [0.34; 1.54]; very low level of evidence) (Fig. 5, Supplementary Table S5).

Adverse events possibly reported to one of the used products were not mentioned in 22 studies^1,21,23,24,26,27,39,40,42–55^ and no (serious) adverse events were observed in 2 studies^25,41^.

### Sensitivity analysis

By excluding studies at high risk of bias^23–26,40–42,52–55^ (or studies using a parallel-arm group design^27,40^) the RR [95%CI] of the comparison with sealing and without additional sealing changed from 0.70 [0.53; 0.93] to 0.85 [0.66; 1.10] (or to 0.70 [0.53; 0.93]), that of the comparison coating materials with and without fluoride release from 0.84 [0.70; 1.01] to 0.83 [0.67; 1.04] (or to 0.82 [0.66; 1.02]) and that of the comparison glass ionomer cement with resin adhesives from 0.72 [0.34; 1.54] to 1.02 [0.82; 1.27] (or unchanged) (Supplementary Fig. 1–5).

## Discussion

The present review investigated the preventive efficacy of different sealants and bonding materials. Twenty-four studies reporting on 34 agents were retrieved from the literature with the aim of investigating the prevention of the appearance and progression of WSL during treatment with fixed orthodontic appliances. This reflects that establishing a gold-standard therapy for prevention of WSL during multi-bracketed treatment is not simple. However, several authors observed that oral hygiene remained a strong predictor for the initiation of new or the progression of existing WSL^23,39,41,52^. Patients with poor hygiene presented a significantly higher risk. Since insufficient oral hygiene and prolonged biofilm accumulation is one of the main factors for WSL formation, this finding underlines that oral hygiene instructions at the beginning and regular dental check-ups during orthodontic treatment seems to be the first choice in management of orthodontic WSL.

The present meta-analysis revealed that, during orthodontic treatment with fixed appliances, the additional use of a sealant significantly decreased the initiation of WSL when compared to its non-use (Odds ratio (OR) [95%CI] = 0.57 [0.36; 0.90]). This is in line with previous reviews on the use of pit and fissure sealants^56^ and proximal sealants or infiltrates^57^. At 24 months follow-up the use of occlusal resin sealants significantly reduced the incidence than non-use (OR [95%CI] = 0.12 [0.08; 0.19]). Furthermore, after a mean follow-up of 25 months a superior efficacy of sealants (OR [95%CI] = 0.29 [0.18; 0.46]) and infiltration (OR [95%CI] = 0.22 [0.15; 0.33]) over non-invasive treatments, including dietary control, biofilm control or control of de- and re-mineralization, were observed. Thus, for all three indications dental sealants seem to provide a physical diffusion barrier that prevents growth of biofilm at the enamel surface and blocks acid diffusion into the dental hard tissue and, consequently, further mineral loss.

No significant difference could be observed between fluoride-releasing agents and those not releasing fluoride. Interestingly, this could be observed for bonding materials during insertion of brackets, for sealants being applied before or after brackets’ bonding as well as for different materials (GIC vs. resin adhesives). This is also in line with the results on pit and fissure sealants and restorative materials^58^. Fluoride releasing resin-based sealants and restorative materials did not provide statistically significant additional benefit compared to the non-fluoride counterparts.

However, the most important limitation of this review are the study designs being used in the included studies. Twenty studies used a split-mouth design, only 4 studies used a parallel-arm group design^23,27,39,40^. The split-mouth design can be used to decrease the risk of confounding factors and eliminate individual differences, such as the naturally predominate side of patients’ brushing and chewing habits, saliva pH and diet, to detect the effect of the coating agents itself and allow each patient to be their own control^40,43^. Nonetheless, when using a split-mouth design, site- and carry-over effects have to be considered^59^. For instance, fluoride being released from the fluoride-containing agent may be carried across from one side to another showing a falsely higher preventive effect for the non-fluoride materials^60^. Both aspects have been addressed in the present meta-analyses: Since both meta-analyses (almost solely) included split-mouth studies and since none of the split-mouth studies used statistical models being adjusted for split-mouth designs (e.g. no site-effect evaluation) or at least did not describe that those models were used evidence of each comparison was downgraded. Furthermore, for the comparison coating materials with and without fluoride release only one study used a parallel-arm group design, whereas eight studies used a split-mouth design. Thus, a cross-over effect of fluoride to the control teeth via saliva has to be considered when interpreting the present results on fluoride-releasing materials^61^.

The efficacy of fluoride-releasing coating agents also depends on their integrity or durability^25^. Sealants’ durability, for example, is most likely negatively influenced due to its inability to resist mechanical abrasion from toothbrushing and mastication^49^. The durability seems also to be influenced by tooth type, jaw and time since application^62^. Consequently, several studies^21,23,25,40,41,49^ highlighted that a regular re-application of sealants may increase their efficacy. However, sealants were reapplied in only one of the included studies^25^ and retention rates of the sealants were also reported only in one study^49^. In this study^49^ only 50% of the sealants were still present on the tooth surfaces after 3 months. Thus, it still remains unclear if re-application of a fluoride-releasing coating agent may increase its efficacy or not.

Visual inspection detecting WSL is a common and frequently employed method, but it has to be reported that the included studies implemented slightly different criteria for the visual assessment. This may lead to certain performance bias. The most common classifications to assess decalcifications were either a 4-point scale^26,40,41,43,45,48–50^, a 3-point scale^24,44,46,47^ or a 2-point scale^23,39,53^. Four of these studies^21,26,40,41^ used the modified scoring system of Gorelick^3^. However, most of them used their own scoring system^23,24,43,46–48,52^. One study even combined the scoring system of 4 different studies^27^, indicating that the used visual-tactile scoring systems were relatively similar. Nonetheless, although all scores used scales from no visible demineralization to cavitation all scores are somehow subjective^41^ and the slight inconsistent definitions in different WSL scores might have influenced the present results^4^.

The available evidence is additionally limited by the follow-up times. Follow-up times were rather short (median: 12.65 months). Seven studies ended within 6 months and only 3 studies reported outcomes for more than 24 months. However, all these factors were reflected in risk of bias analysis and evidence grading.

In conclusion, In the frame of the current systematic review, no gold standard prevention strategy of WSL during brackets can be established. Based on our meta-analyses the additional use of a sealant significantly decreased the initiation of WSL when compared to untreated group. Furthermore, fluoride releasing coating materials do not seem to be more effective than agents without fluoride release. However, results should be interpreted with caution, due to the overall low number of clinical trials for each of the agents, the relative high number of studies using split-mouth designs and the limiting grade of evidence.

## Supplementary Information

Supplementary Information.
