# Supplementary material for: Efficacy of sealants and bonding materials during fixed orthodontic treatment to prevent enamel demineralization: a systematic review and meta-analysis
Source: Sci Rep. 2021 Aug 16;11:16556. doi: 10.1038/s41598-021-95888-6 (PMC8368161; doi:10.1038/s41598-021-95888-6)
Supplement: Supplementary file 1 — Supplementary Information. [file 41598_2021_95888_MOESM1_ESM.docx]

**Efficacy of sealants and bonding materials during fixed orthodontic treatment to prevent enamel demineralization – A systematic review and meta-analysis**

**R. Kamber^1,*^, H. Meyer-Lueckel^1^, D. Kloukos^2^, C. Tennert^1^, R. J. Wierichs^1^**

^1^ Department of Restorative, Preventive and Pediatric Dentistry, zmk bern, University of Bern, Switzerland

^2^ Department of Orthodontics and Dentofacial Orthopedics, zmk bern, University of Bern, Switzerland

**Short title:** Systematic review of coating materials during fixed orthodontic treatment

**Keywords:** demineralization; white spot lesion; prevention; enamel sealants; brackets; fluoride-containing materials

***Correspondence:**

Dr. med. dent. Rita Kamber

Department of Restorative, Preventive and Pediatric Dentistry,

University of Bern, zmk Bern,

Freiburgstrasse 7,

3010 Bern, Switzerland

Tel.: +41 31 632 25 80

E-mail: rita.kamber@zmk.unibe.ch

**Declaration of Interest:** The authors declare no conflicts of interest.

**Supplementary**

**Supplementary table S1.** Search strategy

| Nr. | Database | Search | Hits |
| --- | --- | --- | --- |
| 1 | Pubmed | ((((((((((orthodon*) OR treatment) OR therapy) OR brace*) OR bracket) OR fixed appliance))) AND ((((((sealant) OR primer) OR adhesive) OR (pit and fissure sealant)) OR bonding))) AND ((((((caries) OR white spot) OR decalcification) OR demineralization) OR ICDAS))) AND (((((((((dmft) OR diagnodent) OR progression) OR index) OR visual*) OR ICDAS) OR Quantitative light-induced fluorescence) OR QLF)) | 715 |
| 2 | CENTRAL | Same | 354 |
| 3 | EMBASE | Same | 612 |
|  | **Sum (with overlap)** | | 1681 |
|  | **Sum (without overlap)** | | 984 |

**Suppelmentary table S2. Exclusion by full-text**

| Author | Reason of exclusion |
| --- | --- |
| Al-Eesa 2017 ^1^ | In vitro study |
| Alencar 2009 ^2^ | In vitro study |
| Atilla 2019 ^3^ | Not relevant |
| Behnan 2010 ^4^ | In vitro study |
| Cain 2006 ^5^ | In vitro study |
| Cantekin 2014 ^6^ | In vitro study |
| Corry 2003 ^7^ | In Vitro study |
| Cosma 2019 ^8^ | No clinical study |
| Dalessandri 2012 ^9^ | No assessment of bonding system |
| Devados 2009 ^10^ | In vitro study |
| Feng 2017 ^11^ | In vitro study |
| Gorton 2003 ^12^ | Not relevant |
| Hess 2011 ^13^ | In vitro study |
| Horiuch 2009 ^14^ | In vitro study |
| Iijima 2012 ^15^ | In vitro study |
| Kim 2018 ^16^ | In vitro study |
| Knösel 2012 ^17^ | In vitro study |
| Knösel 2015 ^18^ | No assessment of demineralization |
| Krokmaz 2019 ^19^ | No fixed appliances |
| Korbmacher 2006 ^20^ | In vitro study |
| Lee 2018 ^21^ | Cross-sectional study |
| Lucchese 2013 ^22^ | Cross-sectional |
| Nascimento 2016 ^23^ | Systematic review |
| Nam 2019 ^24^ | In vitro study |
| n/a 2017 ^25^ | Ongoing clinical trial, not published yet |
| n/a 2018 ^26^ | Ongoing clinical trial, not published yet |
| n/a 2017 ^27^ | Ongoing clinical trial, not published yet |
| n/a 2015 ^28^ | Ongoing clinical trial, not published yet |
| Machicek 2011 ^29^ | In vitro study |
| Mitchell 1992 ^30^ | No clinical study |
| Munjal 2016 ^31^ | No assessment of bonding systems |
| Ogaard 2006 ^32^ | No assessment of bonding system |
| Paschos 2009 ^33^ | In vitro study |
| Pellegrini 2009 ^34^ | No assessment of demineralization |
| Poosti 2013 ^35^ | In vitro study |
| Pourhajibagher 2019 ^36^ | In vitro study |
| Preciado 2017 ^37^ | In vitro study |
| Rahimi 2017 ^38^ | Systematic Review |
| Ramazanzade 2013 ^39^ | In vitro study |
| Staudt 2004 ^40^ | In vitro study |
| Underwood 1989 ^41^ | No full-text |
| Uysal 2011 ^42^ | Not relevant |
| Uysal 2010 ^43^ | Not relevant |
| Uysal 2010 ^44^ | In vitro study |
| Visel 2014 ^45^ | No full-text |
| Wang 2015 ^46^ | In vitro study |
| Yap 2014 ^47^ | In vitro study |
| Yu 2017 ^48^ | In vitro study |
| Zachrisson 1978 ^49^ | No assessment of demineralization |
| Zhang 2016 ^50^ | In vitro study |
| Zope 2016 ^51^ | No assessment of demineralization |

**Supplementary table S3.** Characteristics of included studies

| Study | Design; setting; country, language | Age at baseline [mean age (range)] [years] | No. of patients at baseline | No. of patients at end | Drop-out rate | No of teeth at baseline (per group) | No of teeth at end (per group) | Follow-up [months] | OH status; OH measures | | Type of outcomes | Typ of intervention |
| --- | --- | --- | --- | --- | --- | --- | --- | --- | --- | --- | --- | --- |
| Alabdullah et al. 2017 ^52^ | RCT_sm_; Uni; Canada, Syria; EN | 17.6 (13-25) | 34 | 30 | 12% | 680 (340;3 40) | 600 (300; 300) | 12 | good OH; OH instructions | | - visual (score: 0-3, own score) - DIAGNOdent | non fluoride-containing adhesive resin vs. fluoride containing adhesive resin |
| Alshammari and Sanea 2019 ^53^ | RCT_sm_; Uni; Saudi Arabia; EN | n/a (12-35) | 26 | 23 | 12% | 264 (n/a) | 255 (128; 127) | 6 | n/a | | -DIAGNOdent | ACP-containing adhesive vs. conventional resin adhesive |
| Banks and Richmond 1994 ^54^ | non-RCT_sm_; PP; United Kingdom; EN | n/a | 80 | 80 | 0% | Total: 1182 chem (289; 282) light (306; 305) | Total: 1182 chem (289; 282) light (306; 305) | 3 | n/a | | - visual (score: 0-3, Artun and Bobakken 1986) | viscous chemically cured sealant vs. non-viscous visible light-cured sealant vs. no sealant |
| Banks et al. 1997 ^55^ | non-RCT ; n/a ; United Kingdom ; EN | n/a | 50 | 50 | 0% | 737 (366; 371) | 737 (366; 371) | 16.3 | n/a; OH instructions | | - Visual (EDI – enamel decalcification index, Banks and Richmond 1994) | non fluoride-containing adhesive resin vs. fluoride containing adhesive resin |
| Bechtold et al. 2013 ^56^ | RCT_sm_; Uni; Germany; EN/G | 13.4+-1.7 (n/a) | 40 | 39 | 3% | 790 | 770 | 6 | PI, PBI; OH instructions | | -DIAGNOdent | fluoride-containing sealant vs. no sealant |
| Benham et al. 2009 ^57^ | RCT_sm_; Uni; USA; EN | n/a (11-16) | 60 | 60 | 0% | 573 | 573 | 15-18 | n/a | | -visual (score: 0-4, Ogaard's modificaition of the scoring systems proposed by Gorelick et al. 1982)  -DIAGNOdent | sealant vs. no sealant |
| Chung et al. 1998 ^58^ | RCT_sm_; Uni; United Kingdoms; EN | 13.4 (n/a) | 26 | 26 | 0% | 96 (25; 23; 48) | 94 (2?; 2?; 47) | 1 | no extra fluoride; OH instructions | | -visual (score: 0-2, own score) | fluoride uptake (compomer and resin-modified GIC vs. conventional resin adhesive) |
| Comert and Oz 2020 ^59^ | non-RCT; n/a; Turkey; EN | 14.9+-1.43 and 15.8+-2.86 (n/a) | 60 | 56 | 7% | 1090 (546; 544) | 1090 (546; 544) | 13.29+-4.18 | n/a; OH instructions | | - visual (score: 0-3, Gorelick et al., 1982) -DIAGNOdent | fluoride containing adhesive vs. resin adhesive without fluoride |
| Fornell et al. 2002 ^60^ | RCT_sm_; Uni; Sweden; EN | 14.3 (12.4-18.8) | 40 | 39 | 3% | 434 (218; 216) | n/a | 14.7 | PI, GBI; OH instructions | | -visual (score: n/a, Zachrisson&Zachrisson 1971) | anti-adhesive hydrophobic sealant |
| Gaworski et al. 1999 ^61^ | non-RCT; PP; USA; EN | n/a | 16 | 16 | 0% | 298 (149; 149) | 298 (149; 149) | 12-14 | n/a | | - visual (score: none-slight-significant, own score) | conventional GIC vs. resin adhesive |
| Ghiz et al. 2009 ^62^ | RCT_sm_; Uni; USA; EN | n/a | 25 | 25 | 0% | 469 (236; 233) | 469 (236; 233) | 18.24 | PI; OH instructions | | -visual (score: 0-4, own score) | conventional etch sealant vs. self-etching primer |
| Hammad and Knosel 2016 ^63^ | RCT; Uni; Egypt; EN | 14.57+-2.04 (12-18) | 50 | 42 | 16% | n/a (analysis on patient's level) | n/a (analysis on patient's level) | 12 | good OH, API; OH instructions | | - visual (score: 0-1, own score) | sealant vs. no sealant |
| Heinig and Hartmann 2008 ^64^ | non-RCT; PP; Germany; EN/GE | 15.9 (median) (11.6-39.5) | 78 | 78 | 0% | 5788 surface regions approximately 1447 | 5788 surface regions approximately 1447 | 24 | n/a | | - visual (score:0-3, Artun & Brobakken 1986, Banks & Richmond 1994, Zachrisson and Zachrisson 1971 and Gorelik et al. 1982) | fluoride containing sealant vs. no sealant |
|  |  |  |  |  |  |  |  |  |  | |  |  |
|  |  |  |  |  |  |  |  |  |  | |  |  |
|  |  |  |  |  |  |  |  |  |  | |  |  |
|  |  |  |  |  |  |  |  |  |  | |  |  |
|  |  |  |  |  |  |  |  |  |  | |  |  |
| Study | **Design; setting; country, language** | **Age at baseline [mean age (range)] [years]** | **No. of patients at baseline** | **No. of patients at end** | **Drop-out rate** | **No of teeth at baseline (per group)** | **No of teeth at end (per group)** | **Follow-up [months]** | **OH status; OH measures** | | **Type of outcomes** | **Typ of intervention** |
| Kumar Jena et al. 2015 ^65^ | RCT_sm_; Uni; India; EN | 15.5+-2.75 (12-20) | 40 | 40 | 0% | 480 (240; 240) | 480 (240; 240) | 6 | n/a | | - visual (score 0-3, Ogaard's modification 1989, Gorelik et al. 1982) -DIAGNOdent | varnish vs. no varnish |
| Leizer et al. 2010 ^66^ | non-RCT_sm_; PP; USA; EN | 17.6 (10-40) | 22 | 18 | 18% | 243 | 177 (87;90) | 14.6 | n/a; OH instructions | -visual (score 1-3, Wenderroth et al. 1999, Gaworski et al. 1997, Lee et al. 2018) | | fluoride-releasing sealant vs. sealant without fluoride |
| Marcusson et al 1997 ^67^ | RCT_sm_; Uni; Sweden; EN | 13.7 | 60 | 60 | 0% | 222 (111; 111) | 222 (111; 111) | 12 | n/a; OH instructions | - visual (sorce: 1-3, Geiger et al 1988) | | conventional GIC vs. resin adhesive |
| Millett et al. 1999 ^68^ | non-RCT_sm_; Uni; United Kingdom; EN | 13.4+-0.2 (n/a) | 40 | 40 | 0% | 138 (69; 69) | 138 (69; 69) | 15.3 | good OH; n/a | -visual (score: 0-8 and 0-3, Clarkson and O'Mullane 1989) | | conventional GIC vs. resin adhesive |
| Millett et al. 2000 ^69^ | non-RCT_sm_; Uni; United Kingdom; EN | 14.4 (median) | 45 | 45 | 0% | 426 (213; 213) | 294 (147; 147) | compomer: 17,5 (median) resin adhesive: 18,2 (median) | good OH; OH instructions | - visual (score: 1-3, Geiger et al 1988, Marcusson et al. 1997) | | compomer vs. resin adhesive |
| O'Reilly et al. 2013 ^70^ | non-RCT_sm_; PP; USA; EN | 14.6+-3 (10.1-25.4) | 65 | 62 | 5% | n/a | 344 (185; 186) | 26.7+-5.1 range: 16.6-39.2 | n/a; OH instructions | - visual (score 0-3, Gorelik et al. 1982) | | sealant vs. no sealant |
| Trimpeneers and Dermaut 1996 ^71^ | RCT_sm_; Uni; Belgium; EN | 12.8 (10.8-16.2) | 50 | 50 | 0% | 375 (171; 204) | 375 (171; 204) | 21 | n/a; OH instructions | - 0-1 (presence or absence of WSL) | | fluoride-releasing light cured material vs. chemically cured non mix resin |
| Tufekci et al. 2014 ^72^ | non-RCT_sm_; Uni; USA; EN | n/a (10-20) | 22 | 22 | 0% | 72 (36; 36) | 72 (36; 36) | 3 | good OH, PI, PBI; OH instructions | - visual (4 point scale, 0-1-2-3, Alexander and lpa 2000) | | sealant with fluoride vs.  conventional primer without fluoride |
| Turner 1993 ^73^ | non-RCT_sm_; PP; United Kingdom; EN | 14.36+-3,15 (n/a) | 42 | 42 | 0% | 406 (203; 203) | 406 (164; 164) for some parts n is reported as: 406 (203; 203) | 15.4+-4.7 | good OH, PI, GI; OH instructions | -visual (score: 0-3, own score) | | fluoride containing adhesive vs. conventional adhesive |
| Wenderoth et al. 1999 ^74^ | non-RCT_sm_; Uni; USA; EN | 13.2 (9.25-18.8) | 20 | 20 | 0% | 234 (117; 117) | 225 (112; 113) -9 | 12.75 | good OH, PI, GI; OH instructions | - visual (score: 0-1-3, own score) | | sealant vs. no sealant |
| Zingler et al. 2014 ^75^ | RCT; PP; Germany; EN | n/a (11-15) | 126 | 118 | 6% | 2520 (620; 640; 620; 640) | 2360 (560; 600; 580; 620) | 3 | PI, GI; n/a | - visual (score 0-1, DMFT/DMFS) -plaque index (PIB, TQHI, MAPI)  -gingival index (PBI) | | brushing device and sealer comparison |

*CT* clinical trial; *EN* English; *G* German; GI gingiva index; *GBI* gingival bleeding index; *GIC* glass ionomer cement; *mos* months; *OH* oral hygiene; *CT* clinical trial; *RCT* randomized controlled trial; *PI* plaque index; *PP* private praxis or medical centre; *RCT_co_* crossover randomized clinical trial; *RCT_sm_* split mouth randomized clinical trial

**Supplementary table S4.** Sealants and bonding materials

| **Name** | **Manufacturer** | **Fluoride** | **Category** | **Studies** |
| --- | --- | --- | --- | --- |
| Aegis Ortho | Bosworth Co., Sokie, IL | No | Resin | ^53^ |
| AquaCem | DeTrey, Div., Dentsply Ltd, Wybridge, Surrey, UK | Yes | GIC | ^67^ |
| Assure | Reliance Orthodontic Products Inc, Illinois, USA | No | Resin | ^70^ |
| BisCover LV | Bisco, Schaumburg, Illunois, USA | No | Resin | ^70^ |
| Clinpro XT varnish | 3M Unitek, Monrovia, California, USA | Yes | GIC | ^65^ |
| Consice | 3M Unitek, Monrovia, California, USA | No | Resin | ^73^ |
| Dyract Ortho | DeTrey Dentsply, Kostanz, Germany | Yes | Compomer | ^58,69^ |
| Experimental orthodontic bracket resin K32 | De Trey Dentsply, Konstanz, Germany | Yes | Resin | ^73^ |
| Fuji LC Ortho | GC America Incorporated | Yes | GIC | ^61^ |
| Grengloo | Ormco Corporation, Orange, Calif, USA | No | Resin | ^70^ |
| Ketac-Cem | Espe, Oberbay, Germany | Yes | GIC | ^68^ |
| Lee Insta-Bond | Lee Pharmaceuticals, El Monete, Calif, USA | No | Resin | ^71^ |
| Light Bond^TM^ | Reliance Orthodontic Products Inc, Illinois, USA | Yes | Resin | ^52,56,62,64^ |
| Opal Seal | Ultradent, South Jordan, Utah, USA | Yes | Resin | ^59,72^ |
| Maximum Cure | Reliance Orthodontic Products Inc, Illinois, USA | No | Resin | ^54^ |
| Orthon | Orthon Dental, Inc. Vitoria, B.C., Canada | Yes | Resin | ^71^ |
| Ortho-Solo | Ormco Corporation, Orange, Calif, USA | No | Resin | ^57^ |
| Polymeric tooth coating | 3M Unitek, Monrovia, California, USA | No | Resin | ^60^ |
| ProSeal | Reliance Orthodontic Products, Itsaca, Illinois, USA | Yes | Resin | ^66,75^ |
| Protection Plus sealant | Confi-Dental Products Co, Denver, Colorado, USA | Yes | Resin | ^74^ |
| Protecto | BonaDent, Franfurt am Main, Germany | Yes | Resin | ^56^ |
| Reliance Light Bond | Reliance Orthodontic Products, Itsaca, Illinois, USA | No | Resin | ^61^ |
| (standard) Rely-a-Bond | Reliance Orthodontic Products, Itsaca, Illinois, USA | No | Resin | ^55^ |
| (fluoride releasing) Rely-a-Bond | Reliance Orthodontic Products, Itsaca, Illinois, USA | Yes | Resin | ^74^ |
| Right-On | TP Orthodontics, Inc., LaPorte, Indiana, USA | No | Resin | ^54,58,68,69^ |
| SeLECT Defense^TM^ sealant | Lubbock, TX, USA | No | Resin | ^63^ |
| Transbond^TM^ | 3M Unitek, Monrovia, California, USA | No | Resin | ^54,60,64,70^ |
| Transbond MIP | 3M Unitek, Monrovia, California, USA | No | Resin | ^66^ |
| Transbond Plus | 3M Unitek, Monrovia, California, USA | No | Resin | ^62,70^ |
| Transbond Plus L-Pop Seal-Etching Primer | 3M Unitek, Monrovia, California, USA | No | Resin | ^57^ |
| Transbond XT^TM^ | 3M Unitek, Monrovia, California, USA | No | Resin | ^52,53,57,59,63,65,70,72,75^ |
| Ultraseal XT Plus clear sealant | Ultradent Products, South Jordon, Utah, USA | No | Resin | ^57^ |
| Unite | 3M Unitek, Monrovia, California, USA | No | Resin | ^67^ |
| Vitremer | 3M Dental Products Devision, St. Paul, MN, USA | Yes | GIC | ^58^ |

**Supplementary table S5.** Grading of evidence

| **Quality assessment** | | | | | | | **No of patients** | | **Effect** | | **Quality** | **Importance** |  |
| --- | --- | --- | --- | --- | --- | --- | --- | --- | --- | --- | --- | --- | --- |
|  |  |  |  |  |  |  |  |  |  |  |  |  |  |
| **No of studies** | **Design** | **Risk of bias** | **Inconsistency** | **Indirectness** | **Imprecision** | **Other considerations** |  | **Control** | **Relative (95% CI)** | **Absolute** |  |  |  |
| **sealant vs. no sealant (follow-up 3-27 months; assessed with: visual-tactile)** | | | | | | | | | | | | |  |
| 6 | observational studies | serious^1^ | serious^2^ | no serious indirectness | no serious imprecision | reporting bias^3^ | 244/1161  (21%) | 319/1153  (27.7%) | RR 0.7 (0.53 to 0.93) | 83 fewer per 1000 (from 19 fewer to 130 fewer) | ⊕OOO VERY LOW |  |  |
| **coating with fluoride release vs. coating without fluoride release (follow-up 1-24 months; assessed with: visual-tactile)** | | | | | | | | | | | | |  |
| 9 | observational studies | serious^1^ | serious^2^ | no serious indirectness | no serious imprecision | reporting bias^4^ | 568/1980  (28.7%) | 612/1988  (30.8%) | RR 0.84 (0.69 to 1.02) | 49 fewer per 1000 (from 95 fewer to 6 more) | ⊕OOO VERY LOW |  |  |

^1^ see risk of bias assessment
^2^ I^2^ value is large
^3^ 5 studies used a split-mouth design
^4^ 8 studies used a split-mouth design

**Supplemetary figure S1**

Meta-analysis for the comparison: sealant vs. no sealant without studies at high risk of bias. Visual-tactile assessments were used to calculate RR and 95%CI.


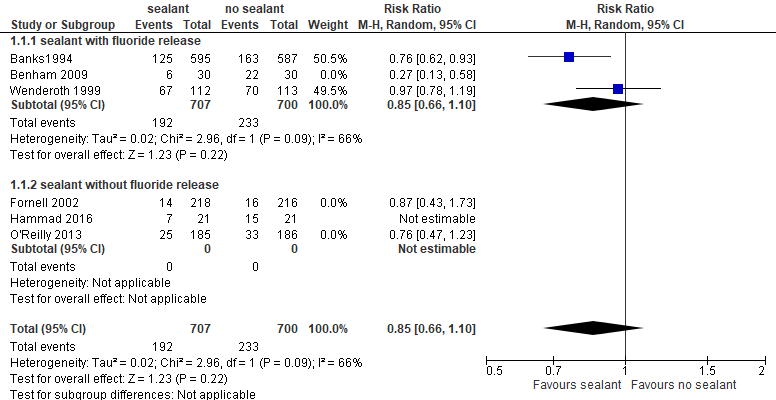


**Supplemenary figure S2**

Meta-analysis for the comparison: sealant vs. no sealant without studies using a parallel-arm design. Visual-tactile assessments were used to calculate RR and 95%CI.


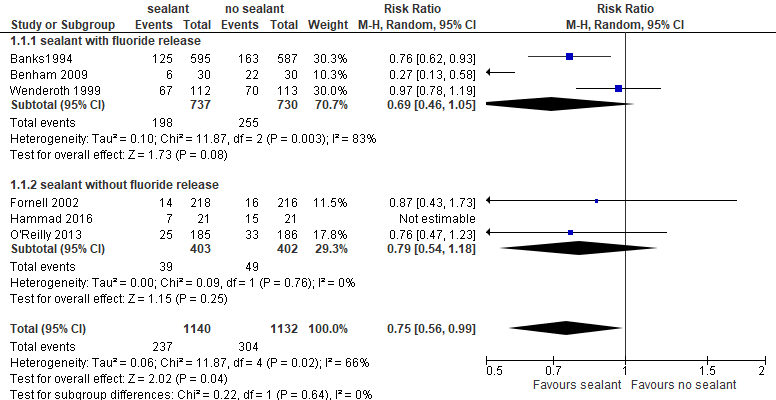


**Supplementary figure S3**

Meta-analysis for the comparison: coating with and without fluoride release without studies at high risk of bias. Visual-tactile assessments were used to calculate RR and 95%CI.


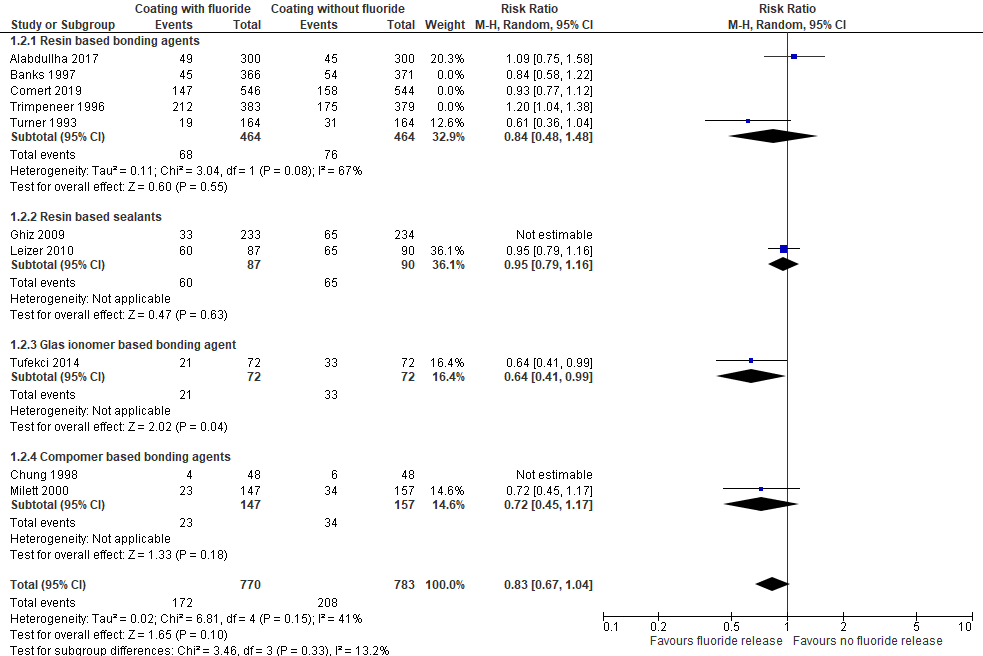


**Supplementary figure S4**

Meta-analysis for the comparison: coating with and without fluoride release studies using a parallel-arm design. Visual-tactile assessments were used to calculate RR and 95%CI.


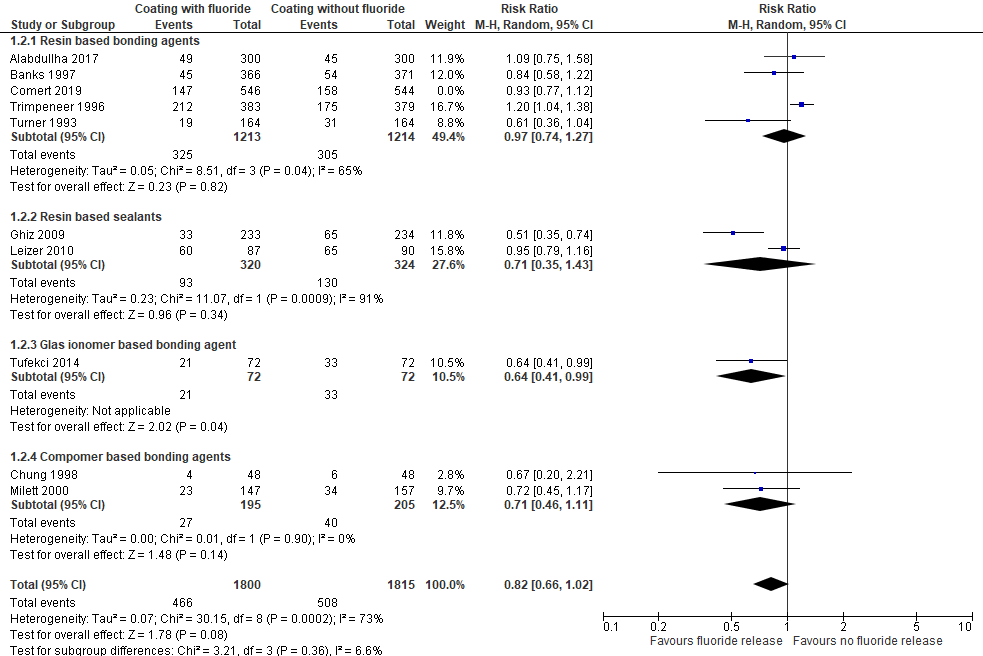


**Supplementary figure S5**

Meta-analysis for the comparison:

glass ionomer cement vs. resin adhesive without studies at high risk of bias, Visual-tactile assessments were used to calculate RR and 95%CI.


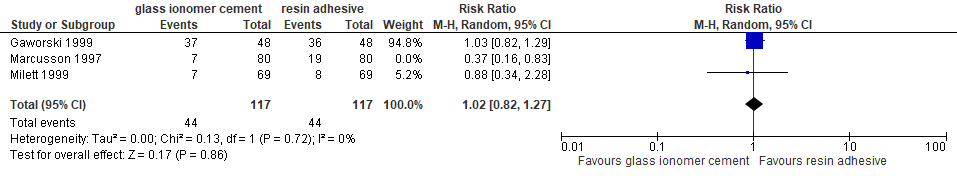


**References**

1 Al-Eesa, N. A., Wong, F. S. L., Johal, A. & Hill, R. G. Fluoride containing bioactive glass composite for orthodontic adhesives - ion release properties. *Dental materials : official publication of the Academy of Dental Materials* **33**, 1324-1329, doi:10.1016/j.dental.2017.08.185 (2017).

2 Alencar, C. J., Braga, M. M., de Oliveira, E., Nicolau, J. & Mendes, F. M. Dye-enhanced laser fluorescence detection of caries lesions around brackets. *Lasers Med Sci* **24**, 865-870, doi:10.1007/s10103-008-0572-0 (2009).

3 Atilla, A. O., Ozturk, T., Eruz, M. M. & Yagci, A. A comparative assessment of orthodontic treatment outcomes using the quantitative light-induced fluorescence (QLF) method between direct bonding and indirect bonding techniques in adolescents: a single-centre, single-blind randomized controlled trial. *Eur J Orthod* **42**, 441-453, doi:10.1093/ejo/cjz058 (2020).

4 Behnan, S. M., Arruda, A. O., Gonzalez-Cabezas, C., Sohn, W. & Peters, M. C. In-vitro evaluation of various treatments to prevent demineralization next to orthodontic brackets. *Am J Orthod Dentofacial Orthop* **138**, 712 e711-717; discussion 712-713, doi:10.1016/j.ajodo.2010.05.014 (2010).

5 Cain, K. *et al.* In vitro enamel caries formation and orthodontic bonding agents. *Am J Dent* **19**, 187-192 (2006).

6 Cantekin, K. & Buyuk, S. K. Shear bond strength of a new low-shrinkage flowable composite for orthodontic bracket bonding. *J Dent Child (Chic)* **81**, 63-66 (2014).

7 Corry, A., Millett, D. T., Creanor, S. L., Foye, R. H. & Gilmour, W. H. Effect of fluoride exposure on cariostatic potential of orthodontic bonding agents: an in vitro evaluation. *J Orthod* **30**, 323-329; discussion 298-329, doi:10.1093/ortho/30.4.323 (2003).

8 Cosma, L. L., Şuhani, R. D., Mesaroş, A. & Badea, M. E. Current treatment modalities of orthodontically induced white spot lesions and their outcome - a literature review. *Med Pharm Rep* **92**, 25-30, doi:10.15386/cjmed-1090 (2019).

9 Dalessandri, D., Dalessandri, M., Bonetti, S., Visconti, L. & Paganelli, C. Effectiveness of an indirect bonding technique in reducing plaque accumulation around braces. *Angle Orthod* **82**, 313-318, doi:10.2319/041811-273.1 (2012).

10 Devados, B. R., Kailasam, V., Padmanabhan, S. & Chitharanjan, A. A comparison of the effects of argon laser and conventional light cure on demineralization resistance of human enamel. *Biomedicine (India)* **29**, 226-230 (2009).

11 Feng, X. *et al.* Novel orthodontic cement containing dimethylaminohexadecyl methacrylate with strong antibacterial capability. *Dent Mater J* **36**, 669-676, doi:10.4012/dmj.2016-370 (2017).

12 Gorton, J. & Featherstone, J. D. In vivo inhibition of demineralization around orthodontic brackets. *Am J Orthod Dentofacial Orthop* **123**, 10-14, doi:10.1067/mod.2003.47 (2003).

13 Hess, E., Campbell, P. M., Honeyman, A. L. & Buschang, P. H. Determinants of enamel decalcification during simulated orthodontic treatment. *Angle Orthod* **81**, 836-842, doi:10.2319/121710-726.1 (2011).

14 Horiuch, S. *et al.* Enamel bonding of self-etching and phosphoric acid-etching orthodontic adhesives in simulated clinical conditions: debonding force and enamel surface. *Dent Mater J* **28**, 419-425, doi:10.4012/dmj.28.419 (2009).

15 Iijima, M. *et al.* Bracket bond strength and cariostatic potential of an experimental resin adhesive system containing Portland cement. *Angle Orthod* **82**, 900-906, doi:10.2319/091311-589.1 (2012).

16 Kim, Y. M. *et al.* Antibacterial and remineralization effects of orthodontic bonding agents containing bioactive glass. *Korean J Orthod* **48**, 163-171, doi:10.4041/kjod.2018.48.3.163 (2018).

17 Knösel, M., Forslund, L., Jung, K. & Ziebolz, D. Efficacy of different strategies in protecting enamel against demineralization during fixed orthodontic treatment. *J Orofac Orthop* **73**, 194-203, doi:10.1007/s00056-012-0072-5 (2012).

18 Knosel, M., Ellenberger, D., Goldner, Y., Sandoval, P. & Wiechmann, D. In-vivo durability of a fluoride-releasing sealant (OpalSeal) for protection against white-spot lesion formation in orthodontic patients. *Head Face Med* **11**, 11, doi:10.1186/s13005-015-0069-6 (2015).

19 Korkmaz, Y. N. & Yagci, A. Comparing the effects of three different fluoride-releasing agents on white spot lesion prevention in patients treated with full coverage rapid maxillary expanders. *Clin Oral Investig* **23**, 3275-3285, doi:10.1007/s00784-018-2749-7 (2019).

20 Korbmacher, H., Huck, L., Adam, T. & Kahl-Nieke, B. Evaluation of an antimicrobial and fluoride-releasing self-etching primer on the shear bond strength of orthodontic brackets. *Eur J Orthod* **28**, 457-461, doi:10.1093/ejo/cjl013 (2006).

21 Lee, S. M. *et al.* Enamel Anti-Demineralization Effect of Orthodontic Adhesive Containing Bioactive Glass and Graphene Oxide: An In-Vitro Study. *Materials (Basel)* **11**, doi:10.3390/ma11091728 (2018).

22 Lucchese, A. & Gherlone, E. Prevalence of white-spot lesions before and during orthodontic treatment with fixed appliances. *Eur J Orthod* **35**, 664-668, doi:10.1093/ejo/cjs070 (2013).

23 Nascimento, P. L., Fernandes, M. T., Figueiredo, F. E. & Faria, E. S. A. L. Fluoride-Releasing Materials to Prevent White Spot Lesions around Orthodontic Brackets: A Systematic Review. *Braz Dent J* **27**, 101-107, doi:10.1590/0103-6440201600482 (2016).

24 Nam, H. J. *et al.* Fluorinated Bioactive Glass Nanoparticles: Enamel Demineralization Prevention and Antibacterial Effect of Orthodontic Bonding Resin. *Materials (Basel)* **12**, doi:10.3390/ma12111813 (2019).

25 DRKS00012540. <https://www.drks.de/drks_web/navigate.do?navigationId=trial.HTML&TRIAL_ID=DRKS00012540>. (2017).

26 NCT03711097. [*https://clinicaltrials.gov/ct2/show/NCT03711097*](https://clinicaltrials.gov/ct2/show/NCT03711097), 2018).

27 IRCT201705099086N3. [*https://www.cochranelibrary.com/central/doi/10.1002/central/CN-01886695/full*](https://www.cochranelibrary.com/central/doi/10.1002/central/CN-01886695/full), 2017).

28 NCT02400957. [*https://clinicaltrials.gov/ct2/show/NCT02400957*](https://clinicaltrials.gov/ct2/show/NCT02400957), 2015).

29 Machicek, S. L. *et al.* Shear bond strengths of a selenium containing and a conventional light cured adhesive for orthodontic bonding. *Tex Dent J* **128**, 1261-1267 (2011).

30 Mitchell, L. Decalcification during orthodontic treatment with fixed appliances--an overview. *Br J Orthod* **19**, 199-205, doi:10.1179/bjo.19.3.199 (1992).

31 Munjal, D., Garg, S., Dhindsa, A., Sidhu, G. K. & Sethi, H. S. Assessment of White Spot Lesions and In-Vivo Evaluation of the Effect of CPP-ACP on White Spot Lesions in Permanent Molars of Children. *J Clin Diagn Res* **10**, ZC149-154, doi:10.7860/JCDR/2016/19458.7896 (2016).

32 Ogaard, B., Alm, A. A., Larsson, E. & Adolfsson, U. A prospective, randomized clinical study on the effects of an amine fluoride/stannous fluoride toothpaste/mouthrinse on plaque, gingivitis and initial caries lesion development in orthodontic patients. *Eur J Orthod* **28**, 8-12, doi:10.1093/ejo/cji075 (2006).

33 Paschos, E., Kurochkina, N., Huth, K. C., Hansson, C. S. & Rudzki-Janson, I. Failure rate of brackets bonded with antimicrobial and fluoride-releasing, self-etching primer and the effect on prevention of enamel demineralization. *Am J Orthod Dentofacial Orthop* **135**, 613-620, doi:10.1016/j.ajodo.2008.01.016 (2009).

34 Pellegrini, P. *et al.* Plaque retention by self-ligating vs elastomeric orthodontic brackets: quantitative comparison of oral bacteria and detection with adenosine triphosphate-driven bioluminescence. *Am J Orthod Dentofacial Orthop* **135**, 426 e421-429; discussion 426-427, doi:10.1016/j.ajodo.2008.12.002 (2009).

35 Poosti, M. *et al.* Shear bond strength and antibacterial effects of orthodontic composite containing TiO2 nanoparticles. *Eur J Orthod* **35**, 676-679, doi:10.1093/ejo/cjs073 (2013).

36 Pourhajibagher, M., Salehi Vaziri, A., Takzaree, N. & Ghorbanzadeh, R. Physico-mechanical and antimicrobial properties of an orthodontic adhesive containing cationic curcumin doped zinc oxide nanoparticles subjected to photodynamic therapy. *Photodiagnosis Photodyn Ther* **25**, 239-246, doi:10.1016/j.pdpdt.2019.01.002 (2019).

37 Preciado, R. M. *et al.* Efficacy of a resin-modified glass ionomer varnish in the prevention of tooth enamel demineralization around orthodontic brackets: An in vitro study. *Investigacion Clinica (Venezuela)* **58**, 365-375 (2017).

38 Rahimi, F., Sadeghi, M. & Mozaffari, H. Efficacy of fluoride varnish for prevention of white spot lesions during orthodontic treatment with fixed appliances: A systematic review study. *BMRAT [Internet]* **4(08)**, 1513-1526. (2017).

39 Ramazanzadeh, B. A., Merati, M., Shafaee, H., Dogon, L. & Sohrabi, K. In-vitro evaluation of an experimental method for bonding of orthodontic brackets with self-adhesive resin cements. *European J Gen Dent* **2**, 264-269, doi:10.4103/2278-9626.116018 (2013).

40 Staudt, C. B., Lussi, A., Jacquet, J. & Kiliaridis, S. White spot lesions around brackets: in vitro detection by laser fluorescence. *Eur J Oral Sci* **112**, 237-243, doi:10.1111/j.1600-0722.2004.00133.x (2004).

41 Underwood, M. L., Rawls, H. R. & Zimmerman, B. F. Clinical evaluation of a fluoride-exchanging resin as an orthodontic adhesive. *Am J Orthod Dentofacial Orthop* **96**, 93-99, doi:10.1016/0889-5406(89)90250-3 (1989).

42 Uysal, T., Amasyali, M., Ozcan, S., Koyuturk, A. E. & Sagdic, D. Effect of antibacterial monomer-containing adhesive on enamel demineralization around orthodontic brackets: an in-vivo study. *Am J Orthod Dentofacial Orthop* **139**, 650-656, doi:10.1016/j.ajodo.2009.06.038 (2011).

43 Uysal, T. *et al.* In vivo effects of amorphous calcium phosphate-containing orthodontic composite on enamel demineralization around orthodontic brackets. *Aust Dent J* **55**, 285-291, doi:10.1111/j.1834-7819.2010.01236.x (2010).

44 Uysal, T., Ustdal, A., Nur, M. & Catalbas, B. Bond strength of ceramic brackets bonded to enamel with amorphous calcium phosphate-containing orthodontic composite. *Eur J Orthod* **32**, 281-284, doi:10.1093/ejo/cjp115 (2010).

45 Visel, D., Jacker, T., Jost-Brinkmann, P. G. & Prager, T. M. Demineralization adjacent to orthodontic brackets after application of conventional and self-etching primer systems. *J Orofac Orthop* **75**, 358-373, doi:10.1007/s00056-014-0233-9 (2014).

46 Wang, X., Wang, B. & Wang, Y. Antibacterial orthodontic cement to combat biofilm and white spot lesions. *Am J Orthod Dentofacial Orthop* **148**, 974-981, doi:10.1016/j.ajodo.2015.06.017 (2015).

47 Yap, J., Walsh, L. J., Naser-Ud Din, S., Ngo, H. & Manton, D. J. Evaluation of a novel approach in the prevention of white spot lesions around orthodontic brackets. *Aust Dent J* **59**, 70-80, doi:10.1111/adj.12142 (2014).

48 Yu, F. *et al.* Antibacterial Activity and Bonding Ability of an Orthodontic Adhesive Containing the Antibacterial Monomer 2-Methacryloxylethyl Hexadecyl Methyl Ammonium Bromide. *Sci Rep* **7**, 41787, doi:10.1038/srep41787 (2017).

49 Zachrisson, B. U. & Brobakken, B. O. Clinical comparison of direct versus indirect bonding with different bracket types and adhesives. *Am J Orthod* **74**, 62-78, doi:10.1016/0002-9416(78)90046-5 (1978).

50 Zhang, N. *et al.* Novel protein-repellent and biofilm-repellent orthodontic cement containing 2-methacryloyloxyethyl phosphorylcholine. *J Biomed Mater Res B Appl Biomater* **104**, 949-959, doi:10.1002/jbm.b.33444 (2016).

51 Zope, A. *et al.* Comparison of Self-Etch Primers with Conventional Acid Etching System on Orthodontic Brackets. *J Clin Diagn Res* **10**, ZC19-ZC22, doi:10.7860/JCDR/2016/18842.9031 (2016).

52 Alabdullah, M. M., Nabawia, A., Ajaj, M. A. & Saltaji, H. Effect of fluoride-releasing resin composite in white spot lesions prevention: a single-centre, split-mouth, randomized controlled trial. *Eur J Orthod* **39**, 634-640, doi:10.1093/ejo/cjx010 (2017).

53 Alshammari, F. M. & Sanea, J. A. Efficacy of Amorphous Calcium Phosphate (ACP) Containing Adhesive in Preventing Demineralization during Orthodontic Treatment, a Triple Blinded Randomized Clinical Trial (RCT). *J Contemp Dent Pract* **20**, 727-731 (2019).

54 Banks, P. A. & Richmond, S. Enamel sealants: a clinical evaluation of their value during fixed appliance therapy. *Eur J Orthod* **16**, 19-25, doi:10.1093/ejo/16.1.19 (1994).

55 Banks, P. A., Burn, A. & O'Brien, K. A clinical evaluation of the effectiveness of including fluoride into an orthodontic bonding adhesive. *Eur J Orthod* **19**, 391-395, doi:10.1093/ejo/19.4.391 (1997).

56 Bechtold, T. E., Sobiegalla, A., Markovic, M., Berneburg, M. & Goz, G. R. In vivo effectiveness of enamel sealants around orthodontic brackets. *J Orofac Orthop* **74**, 447-457, doi:10.1007/s00056-013-0178-4 (2013).

57 Benham, A. W., Campbell, P. M. & Buschang, P. H. Effectiveness of pit and fissure sealants in reducing white spot lesions during orthodontic treatment. A pilot study. *Angle Orthod* **79**, 338-345, doi:10.2319/022808-30.1 (2009).

58 Chung, C. K., Millett, D. T., Creanor, S. L., Gilmour, W. H. & Foye, R. H. Fluoride release and cariostatic ability of a compomer and a resin-modified glass ionomer cement used for orthodontic bonding. *J Dent* **26**, 533-538, doi:10.1016/s0300-5712(98)00017-7 (1998).

59 Comert, S. & Oz, A. A. Clinical effect of a fluoride-releasing and rechargeable primer in reducing white spot lesions during orthodontic treatment. *Am J Orthod Dentofacial Orthop* **157**, 67-72, doi:10.1016/j.ajodo.2019.06.013 (2020).

60 Fornell, A. C., Skold-Larsson, K., Hallgren, A., Bergstrand, F. & Twetman, S. Effect of a hydrophobic tooth coating on gingival health, mutans streptococci, and enamel demineralization in adolescents with fixed orthodontic appliances. *Acta Odontol Scand* **60**, 37-41, doi:10.1080/000163502753471989 (2002).

61 Gaworski, M., Weinstein, M., Borislow, A. J. & Braitman, L. E. Decalcification and bond failure: A comparison of a glass ionomer and a composite resin bonding system in vivo. *Am J Orthod Dentofacial Orthop* **116**, 518-521, doi:10.1016/s0889-5406(99)70182-4 (1999).

62 Ghiz, M. A., Ngan, P., Kao, E., Martin, C. & Gunel, E. Effects of sealant and self-etching primer on enamel decalcification. Part II: an in-vivo study. *Am J Orthod Dentofacial Orthop* **135**, 206-213, doi:10.1016/j.ajodo.2007.02.060 (2009).

63 Hammad, S. M. & Knosel, M. Efficacy of a new sealant to prevent white spot lesions during fixed orthodontic treatment : A 12-month, single-center, randomized controlled clinical trial. *J Orofac Orthop* **77**, 439-445, doi:10.1007/s00056-016-0052-2 (2016).

64 Heinig, N. & Hartmann, A. Efficacy of a sealant : study on the efficacy of a sealant (Light Bond) in preventing decalcification during multibracket therapy. *J Orofac Orthop* **69**, 154-167, doi:10.1007/s00056-008-0741-6 (2008).

65 Kumar Jena, A., Pal Singh, S. & Kumar Utreja, A. Efficacy of resin-modified glass ionomer cement varnish in the prevention of white spot lesions during comprehensive orthodontic treatment: a split-mouth study. *J Orthod* **42**, 200-207, doi:10.1179/1465313315Y.0000000005 (2015).

66 Leizer, C., Weinstein, M., Borislow, A. J. & Braitman, L. E. Efficacy of a filled-resin sealant in preventing decalcification during orthodontic treatment. *Am J Orthod Dentofacial Orthop* **137**, 796-800, doi:10.1016/j.ajodo.2008.11.025 (2010).

67 Marcusson, A., Norevall, L. I. & Persson, M. White spot reduction when using glass ionomer cement for bonding in orthodontics: a longitudinal and comparative study. *Eur J Orthod* **19**, 233-242, doi:10.1093/ejo/19.3.233 (1997).

68 Millett, D. T., Nunn, J. H., Welbury, R. R. & Gordon, P. H. Decalcification in relation to brackets bonded with glass ionomer cement or a resin adhesive. *Angle Orthod* **69**, 65-70, doi:10.1043/0003-3219(1999)069<0065:DIRTBB>2.3.CO;2 (1999).

69 Millett, D. T. *et al.* A comparative clinical trial of a compomer and a resin adhesive for orthodontic bonding. *Angle Orthod* **70**, 233-240, doi:10.1043/0003-3219(2000)070<0233:ACCTOA>2.0.CO;2 (2000).

70 O'Reilly, M. T., De Jesus Vinas, J. & Hatch, J. P. Effectiveness of a sealant compared with no sealant in preventing enamel demineralization in patients with fixed orthodontic appliances: a prospective clinical trial. *Am J Orthod Dentofacial Orthop* **143**, 837-844, doi:10.1016/j.ajodo.2013.01.021 (2013).

71 Trimpeneers, L. M. & Dermaut, L. R. A clinical evaluation of the effectiveness of a fluoride-releasing visible light-activated bonding system to reduce demineralization around orthodontic brackets. *Am J Orthod Dentofacial Orthop* **110**, 218-222, doi:10.1016/s0889-5406(96)70112-9 (1996).

72 Tufekci, E., Pennella, D. R., Mitchell, J. C., Best, A. M. & Lindauer, S. J. Efficacy of a fluoride-releasing orthodontic primer in reducing demineralization around brackets: an in-vivo study. *Am J Orthod Dentofacial Orthop* **146**, 207-214, doi:10.1016/j.ajodo.2014.05.016 (2014).

73 Turner, P. J. The clinical evaluation of a fluoride-containing orthodontic bonding material. *Br J Orthod* **20**, 307-313, doi:10.1179/bjo.20.4.307 (1993).

74 Wenderoth, C. J., Weinstein, M. & Borislow, A. J. Effectiveness of a fluoride-releasing sealant in reducing decalcification during orthodontic treatment. *Am J Orthod Dentofacial Orthop* **116**, 629-634, doi:10.1016/s0889-5406(99)70197-6 (1999).

75 Zingler, S. *et al.* A randomized clinical trial comparing the impact of different oral hygiene protocols and sealant applications on plaque, gingival, and caries index scores. *Eur J Orthod* **36**, 150-163, doi:10.1093/ejo/cjt043 (2014).
